# Itaconic Acid: A Regulator of Immune Responses and Inflammatory Metabolism

**DOI:** 10.3390/cimb47070534

**Published:** 2025-07-09

**Authors:** Kai Ma, Pei Zhou, Wei Zhang, Liwu Zeng, Kaixiong Tao, Peng Zhang

**Affiliations:** Department of Gastrointestinal Surgery, Union Hospital, Tongji Medical College, Huazhong University of Science and Technology, Wuhan 430022, China; m202476382@hust.edu.cn (K.M.); zhou2022@hust.edu.cn (P.Z.); zw10240628@163.com (W.Z.); zengliwu0712@163.com (L.Z.)

**Keywords:** immunomodulation, macrophages, SDH, *IRG1*, Nrf2, NLRP3

## Abstract

This article reviews the multifaceted roles of itaconate in immune regulation and inflammatory metabolism. Itaconic acid is a dicarboxylic acid with anti-inflammatory, antioxidant, and anti-tumor properties. It is initially produced by the heating decomposition of citric acid and is closely related to the tricarboxylic acid cycle. In immune regulation, itaconate regulates macrophage function through a variety of mechanisms, including metabolic reprogramming, polarization regulation, inhibition of cytokine production, and regulation of oxidative stress. It can also affect the function of T cells and B cells. In terms of inflammatory metabolism, itaconate can regulate the production of inflammatory factors, inhibit the activity of succinate dehydrogenase, and affect cellular energy metabolism and lipid metabolism. Its mechanism of action involves the inhibition of succinate dehydrogenase, covalent modification of proteins, influence on epigenetic modification, and playing a role through the G protein-coupled receptor OXGR1 (Oxoglutarate Receptor 1). Itaconic acid derivatives have shown good effects in anti-inflammation and anti-oxidation and have broad application prospects in clinical treatment, including the treatment of inflammatory diseases, anti-tumor and anti-microbial infection. However, the long-term safety and side effects of itaconic acid as a therapeutic agent still need to be further studied. Future studies will further explore the synthesis and function of itaconic acid in different cell types, its physiological effects in non-inflammatory conditions, and its potential application in clinical treatment in order to develop new therapeutic strategies and improve the treatment effect of chronic inflammatory and metabolism-related diseases.

## 1. Introduction

**Figure 1:**
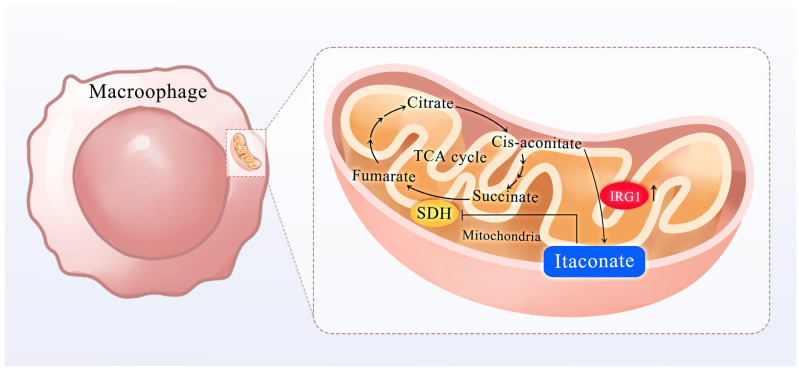
Itaconic acid biosynthesis and its relationship to the tricarboxylic acid cycle.

Itaconic acid, a pivotal dicarboxylic acid, has garnered extensive utilization in polymer synthesis and biomedical applications. Its historical roots extend to the dawn of the 19th century, with its initial isolation achieved through the thermal decomposition of citric acid [1]. Through comprehensive investigations into its biosynthetic pathway, the multifaceted nature of itaconic acid has been progressively elucidated. This versatile compound exhibits a spectrum of biological functions, demonstrating significant pharmacological properties, including anti-inflammatory, antioxidant, and anti-tumor activities [2]. Within biological systems, itaconic acid biosynthesis predominantly occurs in specific fungal species, notably Aspergillus terreus and Ustilago maydis (maize smut fungus), through specialized fermentation processes. These microorganisms possess the metabolic capability to bioconvert citrate precursors into itaconic acid via intricate enzymatic pathways, involving a series of coordinated catalytic reactions [3]. Extensive research has demonstrated that the biosynthesis of itaconic acid is intrinsically linked to the tricarboxylic acid (TCA) cycle, with its production primarily mediated through the enzymatic decarboxylation of cis-aconitic acid, a key intermediate in this metabolic pathway [4]. Furthermore, emerging evidence has revealed that itaconate exerts potent antimicrobial activity within mammalian immune cells, particularly in macrophages, thereby expanding its biological significance in host-pathogen interactions [Figure 1]. Concurrently, the growing emphasis on sustainable chemistry in recent years has driven substantial advancements in itaconic acid production methodologies, particularly in the development of more efficient and environmentally benign fermentation processes. Through metabolic engineering, researchers have enhanced itaconic acid production in Aspergillus, highlighting its industrial potential. Additionally, synthesis using bio-based feedstocks such as citric acid offers new strategies for large-scale production.

Overall, itaconic acid is of significant interest due to its potential in biomedical applications and sustainable processes. As research continues to explore its biosynthetic mechanisms and applications, itaconic acid is poised to play a key role in advancing biotechnology and green chemistry.

## 2. Biosynthesis of Itaconic Acid and Its Inhibitory Effect on Succinate Dehydrogenase

Itaconic acid, a pivotal immunometabolite derived from the tricarboxylic acid (TCA) cycle, is predominantly biosynthesized in activated macrophages via *immune-responsive gene 1 (IRG1)*-mediated pathways [5]. Functioning as a master metabolic regulator, this pleiotropic molecule orchestrates a spectrum of physiological and pathological processes through its unique capacity to simultaneously modulate enzymatic activities and reprogram cellular metabolic networks. Its secretion during macrophage metabolic reprogramming serves as a critical checkpoint for cytokine control under inflammatory conditions, establishing a direct link between immunometabolism and inflammatory responses [6]. The most characterized mechanism involves its potent inhibition of succinate dehydrogenase (SDH), a mitochondrial complex II enzyme essential for both TCA cycle progression and electron transport chain function [7]. By covalently modifying SDH’s catalytic site, itaconate induces succinate accumulation, thereby disrupting oxidative phosphorylation and triggering a metabolic shift toward aerobic glycolysis—a phenomenon now recognized as a hallmark of inflammatory macrophage activation [8,9,10,11].

Beyond its metabolic perturbations, itaconate exerts sophisticated immunomodulatory effects through multiple molecular cascades. Notably, it suppresses pro-inflammatory signaling by directly alkylating cysteine residues on IRAK4 (Interleukin-1 Receptor-Associated Kinase 4), effectively blocking its autophosphorylation and subsequent production of cytokines including TNF-α, IL-6, and IL-1β [12,13]. This dual capacity to regulate both metabolic and inflammatory pathways positions itaconate as a crucial node in the immunometabolic network. Intriguingly, emerging evidence reveals its context-dependent roles in disease pathogenesis: while promoting hepatocellular carcinoma progression through lactate-mediated histone lactylation and metabolic rewiring [14], it conversely enhances osteogenic differentiation and bone formation via epigenetic reprogramming [15]. Such paradoxical effects underscore the complexity of itaconate’s biological functions, which are further modulated by its influence on global ubiquitination patterns and protein post-translational modifications [16]. In addition, it has been shown that the accumulation of the deacetylated metabolite itaconate is closely related to the shift in immune tolerance. In the inflammatory response, the metabolic reprogramming of macrophages increases the production of itaconate, and this metabolic change in turn inhibits SDH activity to regulate the immune response of cells [17].

Therapeutic exploitation of this multifaceted molecule is gaining momentum, particularly for inflammation-associated disorders. Furthermore, the growing appreciation of its tissue-specific effects—from immunomodulation to oncogenesis and bone homeostasis—calls for precision strategies to harness its benefits while mitigating adverse effects. These advances collectively highlight itaconate’s evolution from a simple metabolic intermediate to a central regulator at the crossroads of immunity and metabolism, opening new frontiers for therapeutic intervention in diverse pathological conditions.

## 3. Role of Itaconic Acid in Immune Regulation

### 3.1. Regulation of Macrophage Function by Itaconic Acid and Its Role in Anti-Tumor Immunity

Within the immune response landscape, macrophage functional regulation represents a critical biological process, where itaconate has emerged as a pivotal immunometabolite. Notably, activated macrophages under inflammatory conditions significantly upregulate itaconate production, which subsequently exerts multifaceted regulatory effects on macrophage function through diverse molecular mechanisms [18]. Primarily, itaconate exerts regulatory control over macrophage activity through selective inhibition of intracellular metabolic pathways. Compelling evidence demonstrates that itaconate mediates profound metabolic reprogramming of the tricarboxylic acid (TCA) cycle, resulting in dual functional consequences: modulation of macrophage energy metabolism and alteration of inflammatory response capacity [19,20,21]. Furthermore, itaconate exerts additional regulatory effects on macrophage functionality through its capacity to modulate iron homeostasis and disrupt iron-sulfur cluster stability, thereby influencing crucial cellular processes [22]. Moreover, itaconate serves as a critical modulator of macrophage polarization dynamics. Substantial evidence indicates that itaconate preferentially promotes M2 macrophage polarization, a phenotype associated with anti-inflammatory responses. Mechanistically, itaconate achieves this immunomodulatory effect through a dual mechanism: suppression of M1 macrophage activity and downregulation of pro-inflammatory cytokine production [23,24]. This polarization shift represents a crucial mechanism for modulating both acute and chronic inflammatory responses while facilitating tissue regeneration, positioning itaconate as a promising therapeutic candidate for diverse inflammation-associated pathologies. Importantly, the anti-inflammatory properties of itaconate are further mediated through its regulatory effects on macrophage oxidative stress. Experimental evidence demonstrates that itaconate significantly attenuates intracellular reactive oxygen species (ROS) levels, thereby mitigating oxidative stress-induced cellular damage and maintaining redox homeostasis [25,26]. This cytoprotective effect confers dual benefits: safeguarding macrophage integrity while enhancing their survival and functional capacity under inflammatory conditions.

Itaconic acid has garnered significant attention in recent years for its role in anti-tumor immunity. In addition to its aforementioned functions, itaconic acid can also exert anti-tumor effects by modulating the activity of macrophages [27]. In macrophages, the production of itaconic acid is closely associated with the immune response and is significantly enhanced upon macrophage activation. Following its secretion, itaconic acid enhances the antioxidant capacity of cells by regulating the nuclear factor-erythroid 2-related factor 2 (Nrf2) pathway [28,29]. This mechanism enables itaconate to serve as a critical regulator of macrophage metabolism and function, thereby modulating the immune landscape of the tumor microenvironment. Moreover, recent studies have demonstrated that itaconic acid secreted by tumor-associated macrophages (TAMs) can be internalized by CD8+ T cells, thereby suppressing their proliferation and cytotoxic functions [30]. Thus, targeting itaconic acid production or function may represent a promising strategy to augment anti-tumor immunity. The anti-tumor effects of itaconic acid are also evident in its ability to modulate the tumor microenvironment. By promoting the polarization of macrophages toward the M1 phenotype, itaconic acid enhances the activity of tumor-specific T cells, thereby augmenting their capacity to eliminate tumor cells [31]. This transformation not only enhances anti-tumor immune responses but also potentially inhibits tumor growth and metastasis by modulating the immune cell composition within the tumor microenvironment. Collectively, itaconic acid exerts multifaceted roles in anti-tumor immunity by modulating macrophage function, enhancing the activity of associated immune cells, and remodeling the tumor microenvironment. These findings highlight itaconic acid as a novel concept and a potential therapeutic target in tumor immunotherapy.

Collectively, these findings establish itaconate as a multifaceted regulator of macrophage biology, orchestrating its effects through an integrated network of mechanisms encompassing metabolic reprogramming, polarization modulation, cytokine suppression, and oxidative stress regulation. Itaconate also exerts anti-tumor effects, making it a potential therapeutic target in tumor immunotherapy.

### 3.2. Effects of Itaconic Acid on Other Immune Cells Such as T Cells and B Cells

Within the immune microenvironment, itaconate has emerged as a pivotal immunometabolite that exerts profound regulatory effects on diverse immune cell populations. Notably, its immunomodulatory functions are particularly evident in the regulation of both T cell and B cell activities, highlighting its broad therapeutic potential in immune-related disorders [32]. Accumulating evidence demonstrates that itaconate modulates immune cell activity and functionality through dual regulatory mechanisms: metabolic reprogramming and selective modulation of key signaling transduction pathways. Notably, itaconate exerts particularly profound effects on T cell biology through a multifaceted mechanism. It not only suppresses M2 macrophage polarization but also directly attenuates JAK1 kinase activity, consequently disrupting IL-4 and IL-13 mediated signaling cascades. This integrated regulatory network ultimately leads to the suppression of T cell activation and proliferation [33,34]. Interestingly, itaconate has been shown to enhance, rather than suppress, the effector functions of both CD4+ and CD8+ T cells. This paradoxical effect was particularly evident in vaccination studies using the Tularensis vaccine, where itaconate-deficient mice exhibited enhanced resistance to secondary infections. This protective effect was mechanistically linked to increased T cell populations and augmented functional capacity [35]. Regarding B cell biology, itaconate demonstrates significant immunomodulatory capabilities. Experimental evidence reveals that itaconate positively regulates B cell proliferation and enhances antibody production, particularly through its synergistic effects in T cell-B cell interactions [36,37]. Mechanistically, itaconate potentiates antibody production through a dual mechanism: augmenting B cell activation status and facilitating their differentiation into antibody-secreting plasma cells [38]. Furthermore, itaconate modulates B cell-mediated immune responses through its regulatory effects on cytokine secretion profiles, thereby influencing both humoral immunity and immune cell crosstalk [39]. Notably, the immunomodulatory effects of itaconate extend beyond T and B cells to encompass other immune cells as well. For instance, itaconic acid mitigates chronic inflammation by suppressing macrophage activation and decreasing the secretion of pro-inflammatory cytokines [40]. This mechanism of action suggests that itaconate may hold potential as a therapeutic agent for autoimmune and chronic inflammatory diseases. In conclusion, itaconic acid modulates the functions of T and B cells via multiple mechanisms, thereby influencing their roles in the immune response.

## 4. Role of Itaconic Acid in Inflammatory Metabolism

### 4.1. Regulatory Effects of Itaconic Acid and Its Derivatives on Inflammatory Factors

As an essential metabolite, itaconic acid also serves as a pivotal regulator of the inflammatory response. Emerging studies have demonstrated that itaconic acid modulates the production of inflammatory mediators via multiple mechanisms, thereby influencing both the immune response and the inflammatory state. Firstly, itaconic acid inhibits the production of pro-inflammatory cytokines, including TNF-α and IL-6, thereby attenuating excessive inflammatory responses [41]. In a murine model, itaconic acid derivatives significantly attenuate the levels of the aforementioned cytokines, thereby demonstrating their potential anti-inflammatory efficacy [42,43]. In addition, itaconate affects the inflammatory response by regulating metabolic pathways, specifically by inhibiting succinate dehydrogenase activity, thereby reducing succinate accumulation and the transmission of pro-inflammatory signals [44]. This metabolic reprogramming enables macrophages to more efficiently transition to an anti-inflammatory phenotype during inflammatory states, thereby facilitating tissue repair. Itaconic acid enhances cellular resistance to oxidative stress by binding to kelch-like ECH-associated protein 1 (Keap1) and stabilizing and activating Nrf2 [45,46]. This mechanism not only mitigates inflammation but also safeguards cells against damage.

**Figure 2:**
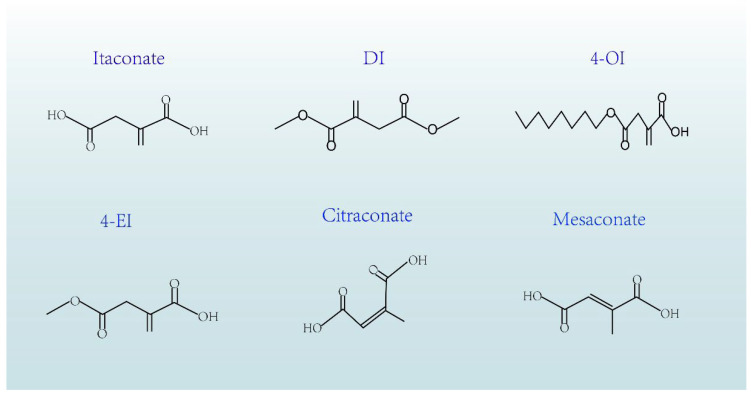
Shown are the chemical structures of itaconate, derivatives, and isomers, including 4-octyl itaconate (4-OI), 4-ethyl itaconate (4-EI), dimethyl itaconate (DI), citraconate, and mesaconate. 4-OI significantly improves survival rates and reduces inflammatory mediators in sepsis models. 4-EI activates the Nrf2 pathway, exerting neuroprotective effects in neurodegenerative disease models. DI modulates immunometabolism and improves insulin resistance in metabolic disorders. Citraconate and mesaconate play key roles in macrophage immunometabolism.

Itaconic acid derivatives have made important breakthroughs in the fields of immune regulation and disease treatment. In clinical studies, itaconate and its derivatives, such as 4-octyl itaconate (4-OI), have demonstrated robust anti-inflammatory effects and can attenuate inflammatory responses associated with various diseases [Figure 2]. For example, 4-OI has significantly improved survival rates and reduced levels of inflammatory mediators in sepsis models [47,48]. These findings provide a strong rationale for itaconate as a potential anti-inflammatory therapeutic agent.

Itaconate modulates the production of inflammatory factors via multiple mechanisms, thereby highlighting its significant potential for immunomodulatory and anti-inflammatory therapies. These studies offer novel insights for the development of future therapeutic strategies targeting inflammation-related diseases.

### 4.2. Anti-Inflammatory Effects of Itaconic Acid in Sepsis

Sepsis is a systemic inflammatory response syndrome characterized by immune dysregulation and multiple organ dysfunction, often resulting from infection [49]. It induces an inflammatory storm and immunosuppression, resulting in multiorgan failure. 4-OI has been reported to significantly improve survival and prevent LPS-induced lethality [50]. Mechanistically, 4-OI has been reported to inhibit ROS production by activating the Nrf2/HO-1 (Heme Oxygenase-1) pathway, thereby attenuating inflammation in mice [51]. As mentioned above, studies have demonstrated that itaconate functions as an SDH inhibitor. Consequently, it may prevent sepsis-associated lethality by inhibiting succinate oxidation. However, further experiments are required to validate this hypothesis.

**Figure 3:**
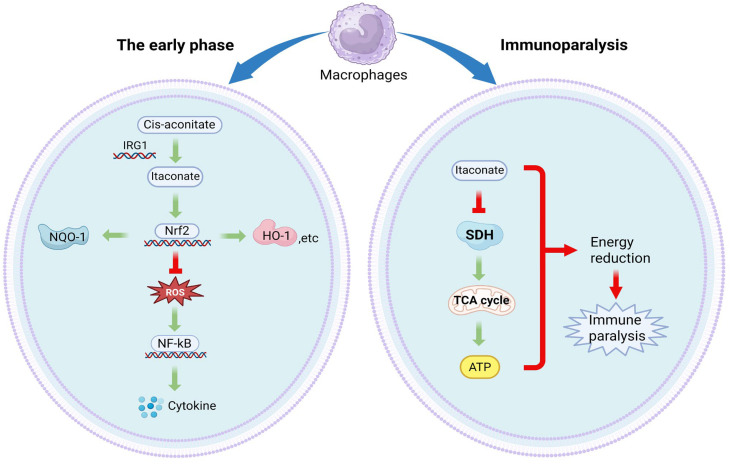
Dual-phase role of Itaconate in sepsis.

As septic disease progresses, the initial hyperinflammatory state transitions to immunosuppression, thereby increasing susceptibility to secondary infections. In the later stages, sepsis induces a state of immunodeficiency in the host, termed sepsis-associated immunosuppression (SAIS). In clinical practice, SAIS frequently results in mortality among septic patients due to secondary infections, characterized by neutrophil and monocyte dysfunction. Elevated *IRG1* expression has been documented in peripheral blood monocytes from septic patients and macrophages from LPS-tolerant mice [52]. Itaconate has been reported to inhibit SDH, thereby attenuating mitochondrial ATP production [53]. This inhibition results in TCA cycle disruption in the late stages of sepsis, ultimately leading to decreased ATP levels. Thus, the pro-inflammatory effects of itaconate in the late stages may be attributed to TCA cycle inhibition and diminished energy production. Moreover, β-glucan has been reported to upregulate SDH expression and downregulate *IRG1* expression in human monocytes, thereby maintaining TCA cycle integrity and restoring immune function [54]. Thus, appropriately inhibiting itaconate production during the immuno-paralytic phase may help control sepsis progression [Figure 3].

(A) Early Hyperinflammatory Phase:
  Pathogen Recognition: LPS or other pathogen-associated molecular patterns activate macrophages, upregulating *IRG1* and triggering itaconate synthesis from cis-aconitate in the TCA cycle.
  Nrf2/HO-1 Activation: Itaconate (or its derivative, 4-OI) modifies KEAP1, releasing Nrf2 to translocate into the nucleus. This upregulates HO-1 and NQO-1 (NAD(P)H:quinone oxidoreductase 1) to reduce oxidative stress.
  Nrf2 activation leads to subsequent inhibition of NF-κB-mediated cytokine production.
(B) Immunoparalysis Phase:
  Persistent SDH inhibition disrupts the TCA cycle, reducing ATP production.
  Energy depletion impairs immune cell function, leading to immunosuppression.

### 4.3. Effect of Itaconic Acid on Inflammatory Metabolic Pathways

In the inflammatory response, the reprogramming of metabolic pathways is an important biological process, and itaconate, as a key metabolite, plays an important regulatory role. Itaconate not only plays a role in anti-inflammatory responses but also affects the production of inflammatory mediators by regulating the REDOX state and ionization pressure within the cell. Previous studies have reported that itaconic acid can selectively regulate the transcriptional response of cells to inflammatory stimuli by reacting with glutathione to induce ionization pressure in cells [55,56]. In addition, itaconate has also been found to inhibit the induction of IκBζ(Inhibitor of κB Zeta), which is closely related to the mediation of Activating Transcription Factor 3 (ATF3) [57], indicating that itaconate plays an important role in regulating inflammatory signaling pathways.

In the metabolic reprogramming of macrophages, the role of itaconate is not only limited to the inhibition of the inflammatory response but also involves the regulation of cellular energy metabolism. Studies have shown that itaconate reduces mitochondrial ATP production by inhibiting succinate dehydrogenase, thereby affecting the energy status and function of macrophages [58]. This metabolic reprogramming allows macrophages to respond more effectively to pathogens in an inflammatory environment while limiting excessive inflammatory responses and promoting tissue repair. In addition, the anti-inflammatory effect of itaconic acid is also related to its role in regulating lipid metabolism. Itaconic acid can negatively regulate lipid metabolism in the liver, inhibit fat accumulation, and subsequently affect the metabolic state of the whole body [59]. This effect is particularly evident in inflammatory states such as sepsis, and the accumulation of itaconate is closely related to metabolic reprogramming of the liver, suggesting its potential application value in regulating systemic metabolism and inflammatory responses. Taken together, itaconate regulates inflammatory metabolic pathways through various mechanisms, including inhibition of succinate dehydrogenase, regulation of ionization pressure, influence of cellular energy metabolism, and regulation of lipid metabolism.

### 4.4. Effect of Itaconic Acid on Lipid Metabolism

In recent years, itaconic acid has garnered significant attention for its role in modulating lipid metabolism and its potential implications in non-alcoholic fatty liver disease (NAFLD). Studies indicate that *immune response gene 1 (IRG1)*, which encodes the enzyme responsible for itaconic acid production, is highly expressed in diverse cell types, including macrophages and hepatocytes. Importantly, *IRG1*-derived itaconic acid exerts regulatory effects on lipid metabolism through its involvement in immune-inflammatory and metabolic pathways.

The expression level of *immune response gene 1 (IRG1)* is closely associated with hepatic lipid accumulation in the pathogenesis of non-alcoholic fatty liver disease (NAFLD). In *IRG1* knockout (KO) mice fed a high-fat diet (HFD), more severe manifestations of metabolic dysfunction were observed, including exacerbated obesity, dyslipidemia, insulin resistance, and hepatic steatosis, along with significantly elevated serum ALT and AST levels [60]. Mechanistic studies revealed that *IRG1* deficiency dysregulates the expression of key genes involved in lipid uptake, synthesis, and β-oxidation, while concurrently suppressing AKT signaling, a central regulator of metabolic homeostasis [60]. Furthermore, itaconate, the enzymatic product of *IRG1*, ameliorates NAFLD progression by attenuating hepatic triglyceride deposition through direct inhibition of fatty acid synthase (FASN) and acetyl-CoA carboxylase (ACC) activities [52].

Studies have shown that itaconic acid and its derivatives, such as 4-octylitaconic acid (4-OI), can regulate lipid metabolism and inflammatory response through various mechanisms. In terms of lipid synthesis, itaconate not only directly alkylates ATP-citrate lyase (ACLY) to inhibit its activity but also down-regulates the maturation of sterol regulatory element binding protein 1c (SREBP-1c), thereby reducing the production of fatty acids and cholesterol [61,62]. At the same time, itaconate also activates the AMPK signaling pathway, up-regulates the expression of carnitine palmitoyltransferase 1A (CPT1A), and promotes fatty acid β-oxidation [56]. It may enhance the thermogenesis of adipose tissue by inducing uncoupling protein 1 (UCP1) [63].

Notably, the effects of itaconic acid in the liver are not limited to hepatocytes themselves but also involve interactions between macrophages and hepatocytes. Studies have shown that itaconic acid derived from macrophages can affect the metabolic state of hepatocytes in a paracrine manner, enhance their oxidative phosphorylation ability, and thereby reduce lipid accumulation [64]. This process not only involves the regulation of metabolic pathways but may also exert long-term effects through alterations in transcriptional levels.

Although itaconate has shown potential therapeutic value in metabolic diseases such as obesity and atherosclerosis, its effects are concentration dependent and cell specific [65]. Therefore, future studies need to further explore the precise regulatory mechanisms of itaconic acid in different cell types to promote its clinical translation. In general, itaconate has become a new target for the intervention of metabolic diseases by integrating the immune and metabolic regulatory networks. However, its clinical application still faces many challenges and needs further research.

### 4.5. Role of Itaconic Acid in Anemia

As an important immune metabolite, itaconic acid plays a key role in anemia-related diseases. In recent years, studies have shown that itaconate affects heme synthesis and erythropoiesis through a variety of mechanisms, thus playing a regulatory role in the occurrence and development of anemia.

During erythropoiesis, itaconic acid is taken up by erythroid precursors and converted to itconyl-coa, which is a competitive inhibitor of 5-aminolevulinic acid synthase 2 (ALAS2), the rate-limiting enzyme in the heme synthesis pathway. This inhibitory effect leads to a decrease in the synthesis of heme and its precursors, such as protoporphyrin IX (PPIX), thereby inhibiting the maturation and hemoglobulinization of red blood cells. This mechanism provides a new perspective for understanding the Anemia of Inflammation (AI) caused by chronic inflammation [66]. For example, it has been found that under inflammatory conditions, macrophages synthesize itaconate, which in turn inhibits the synthesis of heme in red blood cell precursors, leading to anemia [67].

As mentioned above, the anti-inflammatory effect of itaconate is also related to the activation of the Nrf2 pathway. Itaconic acid increases the expression of downstream genes with antioxidant and anti-inflammatory abilities by activating the anti-inflammatory transcription factor Nrf2. This mechanism not only protects against inflammation but also plays an important role in regulating erythropoiesis and heme synthesis [56]. For example, it has been shown that itaconate inhibits the production of inflammatory factors by activating Nrf2, thereby reducing the negative effects on erythropoiesis [68].

Burch et al. revealed that glutamine generates succinyl-CoA through α-ketoglutarate dehydrogenase, providing a key precursor for heme synthesis [69]. SUGCT (succinyl-CoA:glutarate-CoA transferase) can combine succinyl-coa with itaconic acid to produce itaconyl-CoA and then inhibit the ALAS2 enzyme and block heme synthesis [66]. This indicates that the succinyl-CoA produced by glutamine metabolism not only participates in heme synthesis but also participates in succinic acid metabolism through SUGCT, affecting erythrocyte production. Both of these jointly influence different aspects of heme synthesis and have a synergistic mechanism in the occurrence of anemia. These findings not only deepen our understanding of the pathophysiological mechanism of anemia but also provide potential targets for the development of new therapeutic strategies.

## 5. Mechanism of Action of Itaconic Acid

### 5.1. Itaconic Acid Covalently Modifies Proteins Through a Michael Addition Reaction

The covalent modification of itaconic acid via the Michael addition reaction has emerged as a key strategy in biochemistry and drug development. Itaconic acid is a highly reactive α,β-unsaturated carboxylic acid that readily reacts with sulfhydryl groups in proteins, such as cysteine residues, to form stable covalent conjugates [70]. Such reactions are not only utilized for protein labeling and modification but also employed for targeted drug delivery and bioimaging applications. The Michael addition reaction involves the nucleophilic addition to electron-deficient sites, typically proceeding under mild conditions with high selectivity and efficiency [71]. For example, studies have demonstrated that the Michael addition reaction of itaconic acid with protein sulfhydryl groups can effectively modify protein function, inhibit key glycolytic proteins, and alter their biological activity, thereby exerting negative feedback regulation to counteract inflammation. Moreover, the reactive properties of itaconate render it potentially useful for a broad spectrum of applications in biomaterials and drug development. Studies have also demonstrated that itaconate modification can facilitate the development of novel biosensors and drug carriers capable of releasing drugs in specific biological environments [72], thereby enhancing therapeutic efficacy. In conclusion, the covalent modification of proteins via the Michael addition reaction of itaconic acid offers novel tools and strategies for biomedical research.

### 5.2. Competitive Binding of Itaconic Acid to α-Ketoglutarate Affects Epigenetic Modifications

At the intersection of cellular metabolism and epigenetics, researchers have confirmed that the competitive binding between itaconic acid and α-ketoglutarate (α-KG) significantly impacts epigenetic modifications. As a key intermediate in the tricarboxylic acid (TCA) cycle, α-KG plays a pivotal role in energy metabolism, serves as a cofactor for numerous α-KG-dependent enzymes, and participates in epigenetic processes, including histone demethylation [73]. Studies have demonstrated that α-KG levels directly influence histone acetylation and methylation, thereby modulating gene expression. On the other hand, itaconic acid, an emerging metabolite, has been shown to possess anti-inflammatory properties and regulate immune responses [74]. It alters the cellular metabolic state by inhibiting glycolysis and modulating mitochondrial function, thereby influencing the epigenetic signature of cells. Specifically, itaconic acid can inhibit α-KG-dependent demethylase activity by competitively binding to α-KG, thereby reducing α-KG availability. This may lead to histone hypermethylation and subsequent changes in gene expression [75]. Moreover, itaconate accumulation is closely associated with cellular metabolic reprogramming, particularly in the tumor microenvironment. Under hypoxic conditions, cells often undergo metabolic shifts that reduce α-KG production, a process that may be further exacerbated by elevated itaconate levels [76]. This metabolic reprogramming not only impacts cellular energy metabolism but also influences cell fate and function by modulating the epigenetic state. Thus, the competitive binding between itaconate and α-KG is not only a critical component of metabolic regulation but also a key to elucidating how cells regulate epigenetic states via metabolic pathways.

### 5.3. Itaconic Acid Acts as a Paracrine Signaling Molecule Through the G Protein-Coupled Receptor OXGR1

As an important paracrine signaling molecule, itaconic acid exerts its biological functions via the G protein-coupled receptor oxoglutarate receptor 1 (OXGR1). OXGR1 is an acid-sensitive G protein-coupled receptor that senses changes in the intracellular environment and regulates cellular physiological responses [77]. Studies have demonstrated that the mechanism of action of itaconate is closely associated with its activation of OXGR1, which can initiate multiple intracellular signaling pathways, thereby influencing cell proliferation, migration, and inflammatory responses [78]. The paracrine effect of itaconic acid enables it to modulate cell-cell interactions within the local microenvironment. For example, itaconate promotes tumor cell proliferation and migration via OXGR1 activation in the tumor microenvironment [79,80], similar to its effects in other physiological and pathological contexts. Moreover, OXGR1 activation can further enhance cell adaptability and viability by modulating the release of endogenous signaling molecules. During inflammatory responses, itaconate inhibits the release of pro-inflammatory cytokines via OXGR1 signaling, thereby exerting anti-inflammatory effects. This mechanism resembles the actions of other G protein-coupled receptors, such as GPR84, which similarly play crucial roles in regulating immune cell function and inflammatory responses [81]. Thus, itaconic acid and its derivatives may represent a novel therapeutic target for treating related diseases via the OXGR1 signaling pathway. In summary, itaconic acid functions as a paracrine signaling molecule that regulates cell-to-cell signaling via OXGR1, thereby influencing diverse physiological and pathological processes. This finding introduces novel concepts regarding cell-to-cell interactions and their roles in disease, thereby laying the groundwork for future therapeutic strategies.

## 6. Biological Roles and Mechanisms of Itaconyl-CoA

Itaconyl-CoA (Itaconyl-coenzyme A), the activated form of itaconate, has recently emerged as a research hotspot due to its pleiotropic roles in immunometabolism and host defense. Itaconyl-CoA orchestrates cellular metabolism and inflammatory responses through unique biochemical mechanisms [5]. Notably, this activation process not only potentiates itaconate’s metabolic regulatory capacity but also enables its participation in covalent protein modifications, thereby expanding its biological repertoire.

The molecular role of SUGCT in itaconyl-CoA synthesis has also attracted extensive attention. As mentioned above, it is believed that itaconic acid can be converted into itaconyl-CoA through the reverse succinyl-CoA synthetase (SCS) reaction. However, recent studies have shown that SUGCT, but not SCS, is the key enzyme for itconyl-CoA synthesis. SUGCT generates itaconyl-CoA by combining succinyl-CoA with itaconic acid. This finding reveals a new link in the metabolic pathway of itaconic acid and provides a new entry point for studying the production of itaconyl-CoA [67]. SUGCT plays a crucial role in the synthesis of itaconoyl-CoA, providing a new research direction for the function of itaconic acid during its formation.

During pathogen infection, Itaconyl-CoA exerts bacteriostatic effects by competitively inhibiting isocitrate lyase (ICL), thereby disrupting the glyoxylate shunt and directly compromising energy metabolism in intracellular pathogens such as Salmonella [82]. Beyond metabolic interference, Itaconyl-CoA exerts antimicrobial effects through covalent modification of pathogen-essential enzymes. Notably, it specifically itaconylates bacterial methylmalonyl-CoA mutase (MCM), disrupting branched-chain amino acid (BCAA) metabolism and consequently suppressing pathogen proliferation [83]. In antiviral defense, Itaconyl-CoA activates the transcription factor Nrf2 to upregulate antioxidant gene expression, thereby mitigating virus-induced oxidative stress. This mechanism has been experimentally validated in both influenza A virus (IAV) and Zika virus (ZIKV) infection models [84]. Emerging evidence reveals that Itaconyl-CoA inhibits gasdermin D (GSDMD) oligomerization, thereby blocking the terminal execution phase of pyroptosis. This novel mechanism demonstrates significant protective effects in both sepsis and autoimmune disease models [85].

Given its pivotal role in immunometabolism, Itaconyl-CoA and its derivatives have emerged as promising therapeutic targets for inflammatory disorders. Notably, Itaconyl-CoA analogs demonstrate neuroprotective effects in neurodegenerative diseases such as Alzheimer’s disease (AD) by suppressing neuroinflammation and potentially slowing disease progression [86]. However, the tissue-specific pharmacokinetics and long-term biological consequences of Itaconyl-CoA remain incompletely characterized, particularly regarding its controversial role in tumor microenvironments (TMEs). While it exhibits anti-tumor effects via succinate dehydrogenase (SDH) inhibition and Nrf2 pathway activation [56], concurrent suppression of type I interferon (IFN-I) responses may paradoxically facilitate tumor immune evasion [79].

## 7. Pharmacological Effects and Clinical Potential of Itaconic Acid

### 7.1. Anti-Inflammatory and Antioxidant Effects of Itaconic Acid Derivatives

Studies demonstrate that Itaconic acid and its derivatives inhibit inflammation and oxidative stress, showing potential for treating related diseases. Firstly, itaconate has been shown to attenuate inflammatory responses by inhibiting the nuclear factor kappa-B (NF-κB) signaling pathway. NF-κB is a key transcription factor that regulates the expression of multiple inflammatory factors, and its activation is closely associated with various chronic inflammatory diseases [87]. By inhibiting NF-κB activation, itaconic acid derivatives significantly reduce the release of TNF-α and other pro-inflammatory cytokines, thereby attenuating the inflammatory response [88]. Moreover, antioxidant activity is another key property of itaconic acid derivatives. Studies have demonstrated that itaconic acid can effectively scavenge reactive oxygen species (ROS) in the body, thereby reducing cell damage caused by oxidative stress [52]. Oxidative stress is closely associated with the development and progression of various diseases, including cardiovascular disease, diabetes, and cancer [89,90,91]. By enhancing cellular antioxidant capacity, itaconic acid derivatives not only protect cells from oxidative stress damage but also improve cell function and viability. In specific experimental studies, cell models treated with itaconic acid derivatives exhibited enhanced anti-inflammatory and antioxidant effects. For example, itaconic acid derivatives demonstrated a promising inhibitory effect in LPS-induced inflammation models by significantly reducing inducible nitric oxide synthase (iNOS), prostaglandin E2 (PGE2), and other inflammatory mediators [92]. These results indicate that itaconic acid and its derivatives hold significant potential for modulating inflammatory responses and oxidative stress.

In summary, studies on itaconic acid derivatives in anti-inflammatory and antioxidant contexts provide a robust theoretical and experimental foundation for developing novel therapeutic agents. These compounds effectively inhibit inflammatory responses while enhancing cellular antioxidant capacity.

### 7.2. The Application Prospect of Itaconic Acid in Clinical Treatment

Due to its crucial role in metabolic regulation and its significant anti-inflammatory and anti-tumor properties, itaconate has emerged as a highly promising therapeutic agent. Firstly, itaconic acid, playing a crucial role in the inflammatory response, has been shown to ameliorate sepsis and psoriasis in animal models [93,94]. Moreover, itaconic acid has been shown to alleviate pathological changes in arthritis models by inhibiting the proliferation and migration of fibroblast-like synoviocytes [95]. This anti-inflammatory effect positions itaconic acid as a potentially valuable therapeutic agent for inflammation-related diseases, including rheumatoid arthritis. Derivatives of itaconic acid, such as 4-octyl itaconic acid, have demonstrated robust protective effects in renal fibrosis by inhibiting the TGF-β/ROS pathway and reducing ROS generation [96]. These findings indicate that itaconic acid and its derivatives are promising candidates for clinical application. In the antimicrobial context, combining itaconic acid with antibiotics enhances efficacy. For example, the combination of itaconic acid and tobramycin demonstrates a significant synergistic effect against Pseudomonas aeruginosa biofilm infections, thereby improving biofilm clearance efficiency [97]. This combination therapy offers a novel solution to antibiotic resistance. Moreover, the production and economic feasibility of itaconic acid are crucial factors influencing its clinical application potential. Utilizing inexpensive biomass raw materials, such as sorghum, for itaconic acid production not only lowers production costs [98] but also introduces new strategies for sustainable development. This economic viability enhances the attractiveness of itaconate for future clinical applications.

In conclusion, the clinical application of itaconic acid holds great promise across multiple domains, including anti-inflammatory, anti-tumor, and antimicrobial therapies. As research into the biological characteristics of itaconic acid deepens and its production technology continues to be optimized, itaconic acid is poised to emerge as a significant therapeutic agent, potentially driving new breakthroughs in clinical medicine.

### 7.3. Safety and Efficacy of Itaconic Acid as a Drug Candidate

In recent years, itaconic acid has garnered considerable attention as an emerging drug candidate molecule, owing to its notable safety and efficacy profiles. Itaconic acid is an endogenous small molecule that exhibits anti-inflammatory and antioxidant properties. Studies have demonstrated that itaconate possesses significant therapeutic potential in diverse inflammatory diseases, particularly in mitigating ischemia-reperfusion injury [99]. Application of itaconate has demonstrated significant neuroprotective effects in animal models. For example, itaconate regulates tricarboxylic acid cycle metabolism by inhibiting mitochondrial complex II, thereby reducing oxidative stress associated with ischemia-reperfusion injury [100]. Moreover, itaconate has been shown to enhance neurological function, attenuate inflammation, and promote neuronal survival in a murine model of ischemic brain injury [101]. The safety profile of itaconic acid has been well established, with itaconic acid and its derivatives demonstrating good tolerance across various animal models and exhibiting no significant toxic effects in multiple studies. For example, dimethylitaconic acid has demonstrated substantial analgesic effects in models of chronic inflammatory pain without inducing significant side effects [102,103]. Moreover, itaconate attenuates inflammation by modulating the NLRP3 inflammasome and IL-1β signaling pathway, further supporting its potential as an anti-inflammatory agent. The pharmacokinetic properties of itaconate have demonstrated favorable bioavailability and tissue distribution in preclinical studies [104]. These properties position itaconate as a promising drug candidate, particularly for treating inflammation- and oxidative stress-related diseases. In summary, itaconate has demonstrated favorable safety and efficacy profiles as a potential therapeutic agent, with notable anti-inflammatory and neuroprotective effects. Future studies will continue to explore its clinical application potential.

## 8. Role of Itaconic Acid in Bacterial and Viral Infections and Autoimmune Diseases

In recent years, the research of itaconic acid in infection and autoimmune diseases has attracted wide attention. Itaconic acid not only has antibacterial properties but also regulates the immune response and affects cell metabolism, thus playing a role in a variety of pathological states. In terms of infection, itaconate was found to be able to inhibit the growth of certain bacteria, such as Salmonella typhimurium and Mycobacterium tuberculosis, mainly by inhibiting their key metabolic enzyme isocitrate lyase [105,106]. This antimicrobial effect makes itaconate a potential anti-infective therapeutic strategy, especially in the context of bacterial infections. Itaconic acid can not only inhibit the growth of bacteria but also fight a variety of viral infections, especially in the infection environment where bacteria and viruses coexist [107]. Itaconic acid exerts its anti-inflammatory effect by regulating the host immune response. Itaconic acid has been shown to inhibit cytokine release in macrophages in mice and humans, thereby mitigating the inflammatory response caused by bacterial infection [108,109]. Therefore, itaconic acid acts not only by directly inhibiting bacterial growth but also by regulating the host immune response and the metabolic pathways of the bacteria to enhance their resistance to infection. In terms of antiviral activity, itaconic acid has shown inhibitory effects on a variety of viruses, including influenza virus and novel coronavirus. Studies have shown that itaconate can inhibit virus replication by enhancing the antiviral response of host cells. Specifically, itaconate enhances cellular resistance to viruses by activating the interferon signaling pathway and promoting the expression of antiviral genes. For example, itaconic acid showed good antiviral activity in an influenza virus infection model, being able to significantly reduce viral load and improve pathological changes in the lungs [110]. In summary, itaconate, as an immune metabolite with anti-inflammatory and antiviral properties, shows its potential in the treatment of bacterial and viral infections.

In autoimmune diseases, the immunomodulatory effects of itaconate are equally significant. Studies have shown that itaconate is able to help modulate immune responses and alleviate symptoms of autoimmune diseases by inhibiting the proliferation of cytotoxic T cells. For example, itaconate inhibits the proliferation of CD8+ tissue-resident memory T cells and promotes their apoptosis by regulating the Jak3/Stat3/P53 signaling pathway, thereby reducing the injury response in autoimmune hepatitis [111]. Itaconic acid plays multiple roles in infections and autoimmune diseases, and its ability to modulate metabolic and immune responses makes it a potential therapeutic target.

## 9. Future Research Directions, Challenges, and Prospects

### 9.1. Non-Inflammatory Functions and Therapeutic Potential

Despite extensive research on its role in inflammation, the physiological functions of itaconic acid under non-inflammatory conditions remain underexplored. Itaconic acid enhances cellular antioxidant capacity by modulating the Nrf2 signaling pathway, protecting cells from oxidative stress damage. This antioxidant property enables itaconate to enhance cellular resistance to environmental stress and maintain intracellular stability under non-inflammatory conditions [112]. Additionally, while itaconate is recognized as an inhibitory factor in inflammatory responses, it can also promote the generation of M2-type macrophages by modulating macrophage polarization under non-inflammatory conditions, thereby enhancing tissue repair and regeneration [113]. This regulatory role is crucial for maintaining tissue homeostasis and function, particularly during tissue recovery after injury. Moreover, its potential impact on metabolic diseases also warrants further investigation, as itaconic acid may play a protective role beyond inflammatory contexts. As an immunometabolic regulator, itaconic acid has emerged as a promising therapeutic target for various inflammatory and metabolic diseases. Its ability to modulate immune cell functions, inhibit inflammation, and promote tissue repair positions it as a key player in both inflammatory and non-inflammatory conditions.

### 9.2. Long-Term Safety and Clinical Application

Studies have demonstrated that itaconate not only functions in acute inflammation but also holds potential for treating chronic diseases. However, with increasing clinical use, evaluating the long-term safety and side effects of itaconate becomes particularly important. Despite the demonstrated safety of itaconic acid in animal experiments, caution is warranted in clinical applications. Studies have shown that itaconate not only exhibits anti-inflammatory properties but also regulates the inflammatory response by inhibiting NLRP3 inflammasome activation [114]. The NLRP3 inflammasome is a critical intracellular protein complex that promotes inflammatory pyroptosis and the secretion of pro-inflammatory cytokines, such as IL-1β and IL-18 [115]. However, itaconate could potentially lead to immunosuppression in certain contexts by inhibiting the NLRP3 inflammasome [116]. Therefore, when itaconate is used for therapeutic purposes, its potential impact on the immune system should be carefully considered, particularly in immunocompromised patients. Additionally, the metabolic properties of itaconate may also influence its safety profile with long-term use. For instance, some studies have shown that itaconate can affect erythropoiesis by altering cellular metabolism, which may potentially lead to anemia and other related issues [67]. The effects of this metabolic remodeling are particularly pronounced in chronic inflammatory states. Therefore, monitoring relevant hematological parameters is essential in patients undergoing long-term itaconic acid therapy. These concerns highlight the need for comprehensive studies to understand its long-term impact on the immune system and metabolism. Future research should focus on elucidating its physiological functions under non-inflammatory conditions, evaluating its long-term safety, and exploring its mechanisms in metabolic and immune regulation. This knowledge will be essential for harnessing its therapeutic potential and developing effective treatments for chronic inflammatory and metabolic diseases.

### 9.3. Summary and Future Perspectives

**Figure 4:**
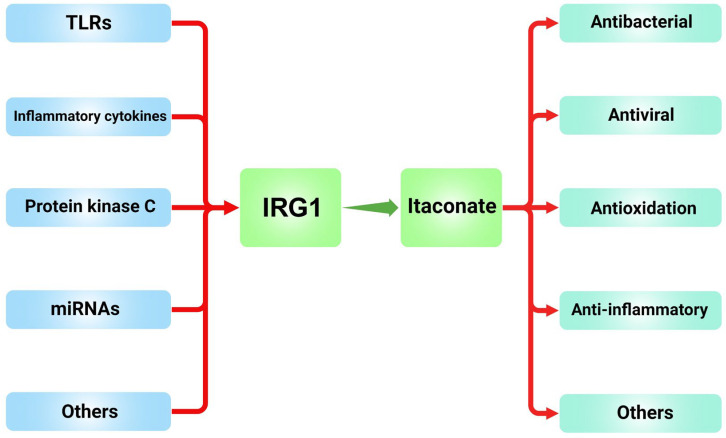
The modulation and role of *IRG1*/itaconate.

Itaconate, functioning as an intermediate in the tricarboxylic acid (TCA) cycle, has been identified as a significant player in the complex interplay between metabolism, immunity, and inflammation. This metabolite’s ability to modulate these interactions is particularly noteworthy as it offers a novel perspective on how the body can be supported in its fight against diseases characterized by immune and inflammatory responses [Figure 4]. To summarize the above, itaconate was found to modulate the inflammatory response by inhibiting the activity of specific enzymes. Additionally, itaconate regulates the activity of Nrf2 through covalent binding to its target proteins within cells, thereby enhancing the antioxidant response. Second, itaconate plays a crucial role in macrophage function. Studies have shown that itaconate can both inhibit the inflammatory response of M1 macrophages and influence the polarization process of M2 macrophages. Furthermore, the anti-inflammatory properties of itaconate position it as a potential therapeutic target. Itaconic acid and its derivatives have demonstrated significant therapeutic efficacy in various models of inflammatory diseases, including the inhibition of bacterial infections and the alleviation of symptoms associated with autoimmune diseases. For example, DI has been shown to induce long-term immune memory and enhance the body’s resistance to infections [117]. Finally, the metabolic properties of itaconate enable it to play a crucial role in regulating the balance between metabolism and immunity in the body. As research on itaconic acid and its derivatives progresses, new therapeutic strategies may emerge to harness their potential for immunometabolic regulation, thereby improving the treatment of chronic inflammatory and metabolism-related diseases. Future research should focus on elucidating its physiological functions under non-inflammatory conditions, evaluating its long-term safety, and exploring its mechanisms in metabolic and immune regulation. This knowledge will be essential for harnessing its therapeutic potential and developing effective treatments for chronic inflammatory and metabolic diseases.

## References

[1] Yu X., Zhang D., Zheng X., Tang C. (2019). Itaconate: An emerging determinant of inflammation in activated macrophages. Immunol. Cell Biol..

[2] Lampropoulou V., Sergushichev A., Bambouskova M., Nair S., Vincent E.E., Loginicheva E., Cervantes-Barragan L., Ma X., Huang S.C., Griss T. (2016). Itaconate links inhibition of succinate dehydrogenase with macrophage metabolic remodeling and regulation of inflammation. Cell Metab..

[3] Ye D., Noh M.H., Moon J.H., Milito A., Kim M., Lee J.W., Yang J.S., Jung G.Y. (2022). Kinetic compartmentalization by unnatural reaction for itaconate production. Nat. Commun..

[4] Chen F., Elgaher W.A.M., Winterhoff M., Büssow K., Waqas F.H., Graner E., Pires-Afonso Y., Casares Perez L., de la Vega L., Sahini N. (2022). Citraconate inhibits ACOD1(IRG1) catalysis, reduces interferon responses and oxidative stress, and modulates inflammation and cell metabolism. Nat. Metab..

[5] Michelucci A., Cordes T., Ghelfi J., Pailot A., Reiling N., Goldmann O., Binz T., Wegner A., Tallam A., Rausell A. (2013). Immune-responsive gene 1 protein links metabolism to immunity by catalyzing itaconic acid production. Proc. Natl. Acad. Sci. USA.

[6] Strelko C.L., Lu W.Y., Dufort F.J., Seyfried T.N., Chiles T.C., Rabinowitz J.D., Roberts M.F. (2011). Itaconic acid is a mammalian metabolite induced during macrophage activation. J. Am. Chem. Soc..

[7] Cordes T., Metallo C.M. (2021). Itaconate alters succinate and coenzyme a metabolism via inhibition of mitochondrial complex II and methylmalonyl-CoA mutase. Metabolites.

[8] Ackermann W.W., Potter V.R. (1949). Enzyme inhibition in relation to chemotherapy. Proc. Soc. Exp. Biol. Med. Soc. Exp. Biol. Med..

[9] Daniels P., Kofman S.B., Smith J.R., Norris G.T., Snyder A.G., Kolb J.P., Gao X., Locasale J.W., Martinez J., Gale M. (2019). The nucleotide sensor ZBP1 and kinase RIPK3 induce the enzyme IRG1 to promote an antiviral metabolic state in neurons. Immunity.

[10] Heinz A., Nonnenmacher Y., Henne A., Khalil M.A., Bejkollari K., Dostert C., Hosseini S., Goldmann O., He W., Palorini R. (2022). Itaconate controls its own synthesis via feedback-inhibition of reverse TCA cycle activity at IDH2. Biochim. Biophys. Acta Mol. Basis Dis..

[11] Shi X., Zhou H., Wei J., Mo W., Li Q., Lv X. (2022). The signaling pathways and therapeutic potential of itaconate to alleviate inflammation and oxidative stress in inflammatory diseases. Redox Biol..

[12] Swain A., Bambouskova M., Kim H., Andhey P.S., Duncan D., Auclair K., Chubukov V., Simons D.M., Roddy T.P., Stewart K.M. (2022). Comparative evaluation of itaconate and its derivatives reveals divergent inflammasome and type I interferon regulation in macrophages. Nat. Metab..

[13] Xia N., Madore V., Albalakhi A., Lin S., Stimpson T., Xu Y., Schwarzschild M.A., Bakshi R. (2023). Microglia-dependent neuroprotective effects of 4-octyl itaconate against rotenone-and MPP^+^-induced neurotoxicity in Parkinson’s disease. Sci. Rep..

[14] Gao X., Pang C., Fan Z., Wang Y., Duan Y., Zhan H. (2024). Regulation of newly identified lysine lactylation in cancer. Cancer Lett..

[15] Sun X., Zhang B., Pan X., Huang H., Xie Z., Ma Y., Hu B., Wang J., Chen Z., Shi P. (2019). Octyl itaconate inhibits osteoclastogenesis by suppressing Hrd1 and activating Nrf2 signaling. FASEB J..

[16] Bourner L.A., Chung L.A., Long H., McGettrick A.F., Xiao J., Roth K., Bailey J.D., Strickland M., Tan B., Cunningham J. (2024). Endogenously produced itaconate negatively regulates innate-driven cytokine production and drives global ubiquitination in human macrophages. Cell Rep..

[17] Jing C., Castro-Dopico T., Richoz N., Tuong Z.K., Ferdinand J.R., Lok L.S.C., Loudon K.W., Banham G.D., Mathews R.J., Cader Z. (2020). Macrophage metabolic reprogramming presents a therapeutic target in lupus nephritis. Proc. Natl. Acad. Sci. USA.

[18] Yang W., Wang Y., Wang T., Li C., Shi L., Zhang P., Yin Y., Tao K., Li R. (2022). Protective effects of IRG1/itaconate on acute colitis through the inhibition of gasdermins-mediated pyroptosis and inflammation response. Genes Dis..

[19] Yin N., Zhang W., Sun X., Wei R., Yang Q., He F., Li C., Guo L., Feng M. (2023). Artificial cells delivering itaconic acid induce anti-inflammatory memory-like macrophages to reverse acute liver failure and prevent reinjury. Cell Rep. Med..

[20] Pan X., Kong X., Feng Z., Jin Z., Wang M., Lu H., Chen G. (2024). 4-Octyl itaconate protects chondrocytes against IL-1β-induced oxidative stress and ferroptosis by inhibiting GPX4 methylation in osteoarthritis. Int. Immunopharmacol..

[21] Zhang S., Jiao Y., Li C., Liang X., Jia H., Nie Z., Zhang Y. (2021). Dimethyl Itaconate alleviates the inflammatory responses of macrophages in sepsis. Inflammation.

[22] Liu X., Shi B., Suo R., Xiong S., Wang X., Liang X., Li X., Li G. (2021). Itaconate regulates macrophage function through stressful iron-sulfur cluster disrupting and iron metabolism rebalancing. FASEB J..

[23] Nakkala J.R., Yao Y., Zhai Z., Duan Y., Zhang D., Mao Z., Lu L., Gao C. (2021). Dimethyl Itaconate-loaded nanofibers rewrite macrophage polarization, reduce inflammation, and enhance repair of myocardic infarction. Small.

[24] An L., Zhai Q., Tao K., Xiong Y., Ou W., Yu Z., Yang X., Ji J., Lu M. (2024). Quercetin induces itaconic acid-mediated M1/M2 alveolar macrophages polarization in respiratory syncytial virus infection. Phytomedicine.

[25] Chen Y., Wang Z., Song Y., Chen N., Guo J., Liu W., Guo K., Ling X., Zhang L. (2023). 4-octyl itaconate improves the viability of D66H cells by regulating the KEAP1-NRF2-GCLC/HO-1 pathway. J. Cell. Mol. Med..

[26] Li W., Li Y., Kang J., Jiang H., Gong W., Chen L., Wu C., Liu M., Wu X., Zhao Y. (2023). 4-octyl itaconate as a metabolite derivative inhibits inflammation via alkylation of STING. Cell Rep..

[27] Zhang X., Qian S., Wu P., Yu B., Yin D., Peng X., Li S., Xiao Z., Xie Z. (2024). Tumor-associated macrophage-derived itaconic acid contributes to nasopharyngeal carcinoma progression by promoting immune escape via TET2. Cell Commun. Signal..

[28] Muri J., Wolleb H., Broz P., Carreira E.M., Kopf M. (2020). Electrophilic Nrf2 activators and itaconate inhibit inflammation at low dose and promote IL-1β production and inflammatory apoptosis at high dose. Redox Biol..

[29] Tang C., Wang X., Xie Y., Cai X., Yu N., Hu Y., Zheng Z. (2018). 4-Octyl Itaconate activates Nrf2 signaling to inhibit pro-inflammatory cytokine production in peripheral blood mononuclear cells of systemic lupus erythematosus patients. Cell. Physiol. Biochem..

[30] Zhao H., Teng D., Yang L., Xu X., Chen J., Jiang T., Feng A.Y., Zhang Y., Frederick D.T., Gu L. (2022). Myeloid-derived itaconate suppresses cytotoxic CD8(+) T cells and promotes tumour growth. Nat. Metab..

[31] Wang J., Long R., Han Y. (2022). The role of exosomes in the tumour microenvironment on macrophage polarization. Biochim. Biophys. Acta Rev. Cancer.

[32] Zhang Z., Chen C., Yang F., Zeng Y.X., Sun P., Liu P., Li X. (2022). Itaconate is a lysosomal inducer that promotes antibacterial innate immunity. Mol. Cell.

[33] McGettrick A.F., O’Neill L.A.J. (2023). Two for the price of one: Itaconate and its derivatives as an anti-infective and anti-inflammatory immunometabolite. Curr. Opin. Immunol..

[34] Runtsch M.C., Angiari S., Hooftman A., Wadhwa R., Zhang Y., Zheng Y., Spina J.S., Ruzek M.C., Argiriadi M.A., McGettrick A.F. (2022). Itaconate and itaconate derivatives target JAK1 to suppress alternative activation of macrophages. Cell Metab..

[35] Roberts L.M., Leighton I., Schwarz B., Wehrly T.D., Evans T.J., Bosio C.M. (2022). Itaconate indirectly influences expansion of effector T cells following vaccination with Francisella tularensis live vaccine strain. Cell. Immunol..

[36] Aso K., Kono M., Kanda M., Kudo Y., Sakiyama K., Hisada R., Karino K., Ueda Y., Nakazawa D., Fujieda Y. (2023). Itaconate ameliorates autoimmunity by modulating T cell imbalance via metabolic and epigenetic reprogramming. Nat. Commun..

[37] O’Neill L.A.J., Artyomov M.N. (2019). Itaconate: The poster child of metabolic reprogramming in macrophage function. Nat. Rev. Immunol..

[38] Wu R., Chen F., Wang N. (2020). ACOD1 in immunometabolism and disease. Cell. Mol. Immunol..

[39] Hooftman A., O’Neill L.A.J. (2019). The immunomodulatory potential of the metabolite itaconate. Trends Immunol..

[40] Li Y., Li B., Xiao X., Qian Q., Wang R., Lyu Z., Chen R., Cui N., Ou Y., Pu X. (2024). Itaconate inhibits CD103^+^ TRM cells and alleviates hepatobiliary injury in mouse models of primary sclerosing cholangitis. Hepatology.

[41] Wu R., Liu J., Wang N., Zeng L., Yu C., Chen F., Wang H., Billiar T.R., Jiang J., Tang D. (2022). Aconitate decarboxylase 1 is a mediator of polymicrobial sepsis. Sci. Transl. Med..

[42] Yang L., Zhou P., Li R., Yin Y., Xie G., Shi L., Zhang P., Tao K. (2024). Investigating the role of itaconate in macrophage activation and oxidative stress injury in sepsis-associated acute kidney injury. Mol. Biol. Rep..

[43] Shao M., Chen J., Zhang F., Su Q., Lin X., Wang W., Chen C., Ren H., Zheng S., Hui S. (2024). 4-Octyl itaconate attenuates renal tubular injury in db/db mice by activating Nrf2 and promoting PGC-1α-mediated mitochondrial biogenesis. Ren. Fail..

[44] Liao S., Han C., Xu D., Fu X., Wang J., Kong L. (2019). 4-Octyl itaconate inhibits aerobic glycolysis by targeting GAPDH to exert anti-inflammatory effects. Nat. Commun..

[45] Song H., Xu T., Feng X., Lai Y., Yang Y., Zheng H., He X., Wei G., Liao W., Liao Y. (2020). Itaconate prevents abdominal aortic aneurysm formation through inhibiting inflammation via activation of Nrf2. EBioMedicine.

[46] Yang W., Wang Y., Zhang P., Sun X., Chen X., Yu J., Shi L., Yin Y., Tao K., Li R. (2022). Immune-responsive gene 1 protects against liver injury caused by concanavalin A via the activation Nrf2/HO-1 pathway and inhibition of ROS activation pathways. Free Radic. Biol. Med..

[47] Yang W., Wang Y., Huang Y., Wang T., Li C., Zhang P., Liu W., Yin Y., Li R., Tao K. (2024). Immune Response Gene-1 [IRG1]/itaconate protect against multi-organ injury via inhibiting gasdermin D-mediated pyroptosis and inflammatory response. Inflammopharmacology.

[48] Chen M., Su W., Chen F., Lai T., Liu Y., Yu D. (2022). Mechanisms underlying the therapeutic effects of 4-octyl itaconate in treating sepsis based on network pharmacology and molecular docking. Front. Genet..

[49] Zhao S., Wu W., Liao J., Zhang X., Shen M., Li X., Lin Q., Cao C. (2022). Molecular mechanisms underlying the renal protective effects of coenzyme Q10 in acute kidney injury. Cell Mol Biol Lett..

[50] Xin Y., Zou L., Lang S. (2021). 4-Octyl itaconate (4-OI) attenuates lipopolysaccharide-induced acute lung injury by suppress ing PI3K/Akt/NF-κB signaling pathways in mice. Exp. Ther. Med..

[51] Zhang P., Wang X., Peng Q., Jin Y., Shi G., Fan Z., Zhou Z. (2022). Four-Octyl itaconate protects chondrocytes against H_2_O_2_ induced oxidative injury and attenuates osteoarthritis progression by activating Nrf2 signaling. Oxid. Med. Cell Longev..

[52] Li Y., Zhang P., Wang C., Han C., Meng J., Liu X., Xu S., Li N., Wang Q., Shi X. (2013). Immune responsive gene 1 (IRG1) promotes endotoxin tolerance by increasing A20 expression in macrophages through reactive oxygen species. J. Biol. Chem..

[53] Zhu X., Long D., Zabalawi M., Ingram B., Yoza B.K., Stacpoole P.W., McCall C.E. (2020). Stimulating pyruvate dehydrogenase complex reduces itaconate levels and enhances TCA cycle anabolic bioenergetics in acutely inflamed monocytes. J. Leukoc. Biol..

[54] Domínguez-Andrés J., Novakovic B., Li Y., Scicluna B.P., Gresnigt M.S., Arts R.J., Oosting M., Moorlag S.J.C.F.M., Groh L.A., Zwaag J. (2019). The itaconate pathway is a central regulatory node linking innate immune tolerance and trained immunity. Cell Metab..

[55] Sun D.S., Chang H.H. (2021). Emerging role of the itaconate-mediated rescue of cellular metabolic stress. Tzu Chi Med. J..

[56] Mills E.L., Ryan D.G., Prag H.A., Dikovskaya D., Menon D., Zaslona Z., Jedrychowski M.P., Costa A.S.H., Higgins M., Hams E. (2018). Itaconate is an anti-inflammatory metabolite that activates Nrf2 via alkylation of KEAP1. Nature.

[57] Du Y., Ma Z., Zheng J., Huang S., Yang X., Song Y., Dong D., Shi L., Xu D. (2022). ATF3 positively regulates antibacterial immunity by modulating macrophage killing and migration functions. Front. Immunol..

[58] Li Y., Gong W., Li W., Liu P., Liu J., Jiang H., Zheng T., Wu J., Wu X., Zhao Y. (2023). The IRG1-Itaconate axis: A regulatory hub for immunity and metabolism in macrophages. Int. Rev. Immunol..

[59] Fu L., Liu H., Cai W., Han D., Zhu X., Yang Y., Xie S. (2022). 4-Octyl Itaconate supplementation relieves soybean diet-induced liver inflammation and glycolipid metabolic disorders by activating the Nrf2-Pparγ pathway in juvenile gibel carp. J. Agric. Food Chem..

[60] Zhang X., Zhi Y., Zan X., Fan K., Chen K., Zhao S., Dai X., Li L., Yang Y., Hu K. (2023). Immune response gene 1 deficiency aggravates high fat diet-induced nonalcoholic fatty liver disease via promotion of redox-sensitive AKT suppression. Biochim. Biophys. Acta (BBA)-Mol. Basis Dis..

[61] Cordes T., Wallace M., Michelucci A., Divakaruni A.S., Sapcariu S.C., Sousa C., Koseki H., Cabrales P., Murphy A.N., Hiller K. (2016). Immunoresponsive Gene 1 and Itaconate Inhibit Succinate Dehydrogenase to Modulate Intracellular Succinate Levels. J. Biol. Chem..

[62] Bambouskova M., Gorvel L., Lampropoulou V., Sergushichev A., Loginicheva E., Johnson K., Korenfeld D., Mathyer M.E., Kim H., Huang L.H. (2018). Electrophilic properties of itaconate and derivatives regulate the IκBζ-ATF3 inflammatory axis. Nature.

[63] Qin W., Qin K., Zhang Y., Jia W., Chen Y., Cheng B., Peng L., Chen N., Liu Y., Zhou W. (2019). S-glycosylation-based cysteine profiling reveals regulation of glycolysis by itaconate. Nat. Chem. Biol..

[64] Weiss J.M., Palmieri E.M., Gonzalez-Cotto M., Bettencourt I.A., Megill E.L., Snyder N.W., McVicar D.W. (2023). Itaconic acid underpins hepatocyte lipid metabolism in non-alcoholic fatty liver disease in male mice. Nat. Metab..

[65] Ryan D.G., Murphy M.P., Frezza C., Prag H.A., Chouchani E.T., O’Neill L.A., Mills E.L. (2019). Coupling Krebs cycle metabolites to signalling in immunity and cancer. Nat. Metab..

[66] Medlock A.E., Dailey H.A. (2022). New Avenues of Heme Synthesis Regulation. Int. J. Mol. Sci..

[67] Marcero J.R., Cox J.E., Bergonia H.A., Medlock A.E., Phillips J.D., Dailey H.A. (2021). The immunometabolite itaconate inhibits heme synthesis and remodels cellular metabolism in erythroid precursors. Blood Adv..

[68] Meiser J., Kraemer L., Jaeger C., Madry H., Link A., Lepper P.M., Hiller K., Schneider J.G. (2018). Itaconic acid indicates cellular but not systemic immune system activation. Oncotarget.

[69] Burch J.S., Marcero J.R., Maschek J.A., Cox J.E., Jackson L.K., Medlock A.E., Phillips J.D., Dailey H.A. (2018). Glutamine via alpha-ketoglutarate dehydrogenase provides succinyl-CoA for heme synthesis during erythropoiesis. Blood.

[70] Day E.A., O’Neill L.A.J. (2022). Protein targeting by the itaconate family in immunity and inflammation. Biochem. J..

[71] Xu Y., Li F., Ma J., Li J., Xie H., Wang C., Chen P., Wang L. (2022). Lipase-catalyzed phospha-michael addition reactions under mild conditions. Molecules.

[72] Ding Q., Jing X., Yao S., Su W., Ye B., Qu Y., Gao F., Sun T., Guo X. (2022). Multifunctional hydrogel loaded with 4-octyl itaconate exerts antibacterial, antioxidant and angiogenic properties for diabetic wound repair. Biomater. Adv..

[73] Milanović M., Bekić M., Đokić J., Vučević D., Čolić M., Tomić S. (2024). Exogenous α-ketoglutarate modulates redox metabolism and functions of human dendritic cells, altering their capacity to polarise T cell response. Int. J. Biol. Sci..

[74] Xu L., Cai J., Li C., Yang M., Duan T., Zhao Q., Xi Y., Sun L., He L., Tang C. (2023). 4-Octyl itaconate attenuates LPS-induced acute kidney injury by activating Nrf2 and inhibiting STAT3 signaling. Mol. Med..

[75] Chen L.L., Morcelle C., Cheng Z.L., Chen X., Xu Y., Gao Y., Song J., Li Z., Smith M.D., Shi M. (2022). Itaconate inhibits TET DNA dioxygenases to dampen inflammatory responses. Nat. Cell Biol..

[76] Lang R., Siddique M.N.A.A. (2024). Control of immune cell signaling by the immuno-metabolite itaconate. Front. Immunol..

[77] Wang Q., Zhang Q., Zhang Y., Zhao X. (2019). Yak OXGR1 promotes fibroblast proliferation via the PI3K/AKT pathways. J. Cell. Biochem..

[78] Zeng Y.R., Song J.B., Wang D., Huang Z.X., Zhang C., Sun Y.P., Shu G., Xiong Y., Guan K.L., Ye D. (2023). The immunometabolite itaconate stimulates OXGR1 to promote mucociliary clearance during the pulmonary innate immune response. J. Clin. Investig..

[79] Weiss J.M., Davies L.C., Karwan M., Ileva L., Ozaki M.K., Cheng R.Y., Ridnour L.A., Annunziata C.M., Wink D.A., McVicar D.W. (2018). Itaconic acid mediates crosstalk between macrophage metabolism and peritoneal tumors. J. Clin. Investig..

[80] Sun Y., Li Y., Nan S., Zhang L., Huang H., Wang J. (2015). Synthesis and characterization of pH-sensitive poly(itaconic acid)-poly(ethylene glycol)-folate-poly(l-histidine) micelles for enhancing tumor therapy and tunable drug release. J. Colloid Interface Sci..

[81] Wei L., Tokizane K., Konishi H., Yu H.R., Kiyama H. (2017). Agonists for G-protein-coupled receptor 84 (GPR84) alter cellular morphology and motility but do not induce pro-inflammatory responses in microglia. J. Neuroinflammation.

[82] Naujoks J., Tabeling C., Dill B.D., Hoffmann C., Brown A.S., Kunze M., Kempa S., Peter A., Mollenkopf H.J., Dorhoi A. (2016). IFNs modify the proteome of Legionella-containing vacuoles and restrict infection via IRG1-derived itaconic acid. PLoS Pathog..

[83] Ruetz M., Campanello G.C., Purchal M., Shen H., McDevitt L., Gouda H., Wakabayashi S., Zhu J., Rubin E.J., Warncke K. (2019). Itaconyl-CoA forms a stable biradical in methylmalonyl-CoA mutase and derails its activity and repair. Science.

[84] Olagnier D., Farahani E., Thyrsted J., Blay-Cadanet J., Herengt A., Idorn M., Hait A., Hernaez B., Knudsen A., Iversen M.B. (2020). SARS-CoV2-mediated suppression of NRF2-signaling reveals potent antiviral and anti-inflammatory activity of 4-octyl-itaconate and dimethyl fumarate. Nat. Commun..

[85] Hooftman A., Angiari S., Hester S., Corcoran S.E., Runtsch M.C., Ling C., Ruzek M.C., Slivka P.F., McGettrick A.F., Banahan K. (2020). The immunomodulatory metabolite itaconate modifies NLRP3 and inhibits inflammasome activation. Cell Metab..

[86] Jha A.K., Huang S.C., Sergushichev A., Lampropoulou V., Ivanova Y., Loginicheva E., Chmielewski K., Stewart K.M., Ashall J., Everts B. (2015). Network integration of parallel metabolic and transcriptional data reveals metabolic modules that regulate macrophage polarization. Immunity.

[87] Xu M., Jiang P., Sun H., Yuan X., Gao S., Guo J., Zhao C., Hu X., Liu X., Fu Y. (2020). Dimethyl itaconate protects against lipopolysaccharide-induced endometritis by inhibition of TLR4/NF-κB and activation of Nrf2/HO-1 signaling pathway in mice. Iran. J. Basic Med. Sci..

[88] Ma E., Xing H., Pei J., Zhang Q., Li R., Shen C., Tao Y., Li J., Ma Z., Zhao J. (2022). Itaconic acid facilitates inflammation abatement and alleviates liver ischemia-reperfusion injury by inhibiting NF-κB/NLRP3/caspase-1inflammasome axis. Ann. Transl. Med..

[89] Xu J., Xu S. (2024). The emerging role of ACOD1/itaconate pathway in atherosclerosis. Trends Mol. Med..

[90] He S., Zhao Y., Wang G., Ke Q., Wu N., Lu L., Wu J., Sun S., Jin W., Zhang W. (2023). 4-Octyl itaconate attenuates glycemic deterioration by regulating macrophage polarization in mouse models of type 1 diabetes. Mol. Med..

[91] Gautam A.K., Kumar P., Raj R., Kumar D., Bhattacharya B., Rajinikanth P.S., Chidambaram K., Mahata T., Maity B., Saha S. (2022). Preclinical evaluation of Dimethyl Itaconate against hepatocellular carcinoma via activation of the e/iNOS-Mediated NF-κB-Dependent apoptotic pathway. Front. Pharmacol..

[92] Yang W., Wang Y., Zhang P., Wang T., Li C., Tong X., Zeng X., Yin Y., Tao K., Li R. (2022). Hepatoprotective role of 4-Octyl Itaconate in Concanavalin A-induced autoimmune hepatitis. Mediat. Inflamm..

[93] Zhang P., Wang Y., Yang W., Yin Y., Li C., Ma X., Shi L., Li R., Tao K. (2022). 4-Octyl itaconate regulates immune balance by activating Nrf2 and negatively regulating PD-L1 in a mouse model of sepsis. Int. J. Biol. Sci..

[94] You M., Jiang Q., Huang H., Ma F., Zhou X. (2023). 4-Octyl itaconate inhibits inflammation to attenuate psoriasis as an agonist of oxeiptosis. Int. Immunopharmacol..

[95] Daly R., Blackburn G., Best C., Goodyear C.S., Mudaliar M., Burgess K., Stirling A., Porter D., McInnes I.B., Barrett M.P. (2020). Changes in plasma itaconate elevation in early rheumatoid arthritis patients elucidates disease activity associated macrophage activation. Metabolites.

[96] Zou X., Wu M., Tu M., Tan X., Long Y., Xu Y., Li M. (2024). 4-octyl itaconate inhibits high glucose induced renal tubular epithelial cell fibrosis through TGF-β-ROS pathway. J. Recept. Signal Transduct. Res..

[97] Huang Q., Duan C., Ma H., Nong C., Zheng Q., Zhou J., Zhao N., Mou X., Liu T., Zou S. (2024). Structural and functional characterization of itaconyl-CoA hydratase and citramalyl-CoA lyase involved in itaconate metabolism of *Pseudomonas aeruginosa*. Structure.

[98] Elkasaby T., Hanh D.D., Kahar P., Kawaguchi H., Sazuka T., Kondo A., Ogino C. (2024). Utilization of sweet sorghum juice as a carbon source for enhancement of itaconic acid production in engineered *Corynebacterium glutamicum*. Enzym. Microb. Technol..

[99] Zhu D., Zhao Y., Luo Y., Qian X., Zhang Z., Jiang G., Guo F. (2021). Irg1-itaconate axis protects against acute kidney injury via activation of Nrf2. Am. J. Transl. Res..

[100] Cordes T., Lucas A., Divakaruni A.S., Murphy A.N., Cabrales P., Metallo C.M. (2020). Itaconate modulates tricarboxylic acidand redox metabolism to mitigate reperfusion injury. Mol. Metab..

[101] Song X., Long D. (2020). Nrf2 and Ferroptosis: A new research direction for neurodegenerative diseases. Front. Neurosci..

[102] Nosenko M., Anisov D., Gubernatorova E., Gorshkova E., Zeng Y.R., Ye D., Wang P., Finlay D., Drutskaya M., Nedospasov S. (2024). Itaconate and dimethyl itaconate upregulate IL-6 production in the LPS-induced inflammation in mice. J. Leukoc. Biol..

[103] Lin J., Ren J., Zhu B., Dai Y., Gao D.S., Xia S., Cheng Z., Huang Y., Yu L. (2022). Dimethyl Itaconate attenuates CFA-induced inflammatory pain via the NLRP3/IL-1β signaling pathway. Front. Pharmacol..

[104] Diankristanti P.A., Ng I.S. (2023). Microbial itaconic acid bioproduction towards sustainable development: Insights, challenges, and prospects. Bioresour. Technol..

[105] Ki N., Kim J., Jo I., Hyun Y., Ryu S., Ha N.C. (2022). Isocitrate binds to the itaconic acid-responsive LysR-type transcriptional regulator RipR in Salmonella pathogenesis. J. Biol. Chem..

[106] Nair S., Huynh J.P., Lampropoulou V., Loginicheva E., Esaulova E., Gounder A.P., Boon A.C.M., Schwarzkopf E.A., Bradstreet T.R., Edelson B.T. (2018). Irg1 expression in myeloid cells prevents immunopathology during *M. tuberculosis* infection. J. Exp. Med..

[107] Wu R., Kang R., Tang D. (2022). Mitochondrial ACOD1/IRG1 in infection and sterile inflammation. J. Intensive Med..

[108] Zhang P., Yin Y., Wang T., Li W., Li C., Zeng X., Yang W., Zhang R., Tang Y., Shi L. (2020). Maresin 1 mitigates concanavalin A-induced acute liver injury in mice by inhibiting ROS-mediated activation of NF-κB signaling. Free Radic. Biol. Med..

[109] Bambouskova M., Potuckova L., Paulenda T., Kerndl M., Mogilenko D.A., Lizotte K., Swain A., Hayes S., Sheldon R.D., Kim H. (2021). Itaconate confers tolerance to late NLRP3 inflammasome activation. Cell Rep..

[110] Song J.W., Lam S.M., Fan X., Cao W.J., Wang S.Y., Tian H., Chua G.H., Zhang C., Meng F.P., Xu Z. (2020). Omics-driven systems interrogation of metabolic dysregulation in COVID-19 pathogenesis. Cell Metab..

[111] Zhou P., Tao K., Zeng L., Zeng X., Wan Y., Xie G., Liu X., Zhang P. (2024). IRG1/Itaconate inhibits proliferation and promotes apoptosis of CD69^+^CD103^+^CD8^+^ tissue-resident memory T cells in autoimmune hepatitis by regulating the JAK3/STAT3/P53 signalling pathway. Apoptosis.

[112] Luo Z., Sheng Z., Hu L., Shi L., Tian Y., Zhao X., Yang W., Xiao Z., Shen D., Wu W. (2024). Targeted macrophage phagocytosis by Irg1/itaconate axis improves the prognosis of intracerebral hemorrhagic stroke and peritonitis. EBioMedicine.

[113] Qiu J.H., Zhang L., Li K.X., Zhang Q.H., Fan K.R., Chen K., Jiang Y., Liu G. (2023). Deficiency of IRG1/ itaconate aggravates endotoxemia-induced acute lung injury by inhibiting autophagy in mice. Exp. Anim..

[114] Zhou P., Yang L., Li R., Yin Y., Xie G., Liu X., Shi L., Tao K., Zhang P. (2024). IRG1/itaconate alleviates acute liver injury in septic mice by suppressing NLRP3 expression and its mediated macrophage pyroptosis via regulation of the Nrf2 pathway. Int. Immunopharmacol..

[115] Swanson V., Deng M., Ting J.P. (2019). The NLRP3 inflammasome: Molecular activation and regulation to therapeutics. Nat. Rev. Immunol..

[116] Hoyle C., Green J.P., Allan S.M., Brough D., Lemarchand E. (2022). Itaconate and fumarate derivatives inhibit priming and activation of the canonical NLRP3 inflammasome in macrophages. Immunology.

[117] Ferreira A.V., Kostidis S., Groh L.A., Koeken V.A.C.M., Bruno M., Baydemir I., Kilic G., Bulut Ö., Andriopoulou T., Spanou V. (2023). Dimethyl itaconate induces long-term innate immune responses and confers protection against infection. Cell Rep..

